# COMPLICATIONS REQUIRING HOSPITAL MANAGEMENT AFTER BARIATRIC SURGERY

**DOI:** 10.1590/S0102-6720201500S100003

**Published:** 2015-12

**Authors:** Aline WRZESINSKI, Jéssica Moraes CORRÊA, Tainiely Müller Barbosa FERNANDES, Letícia Fernandes MONTEIRO, Fabiana Schuelter TREVISOL, Ricardo Reis do NASCIMENTO

**Affiliations:** 1 South Santa Catarina University; 2 Nossa Senhora da Conceição Hospital, Pró-Vida Clinic and Socimed Hospital, Tubarão, SC, Brazil

**Keywords:** Morbid obesity, Bariatric surgery, Postoperative complications

## Abstract

**Background:**

The actual gold standard technique for obesity treatment is the Roux-en-Y gastric bypass. However, complications may occur and the surgeon must be prepared for them.

**Aim:**

To evaluate retrospectively the complications occurrence and associated factors in patients who underwent bariatric surgery.

**Methods:**

In this study, 469 medical charts were considered, from patients and from data collected during outpatient consultations. The variables considered were gender, age, height, pre-operatory BMI, pre-operatory weight, pre-operatory comorbidities, time of hospital stay, postoperative complications that demanded re-admission to the hospital and the time elapsed between the procedure and the complication. The patients' follow up was, at least, one year.

**Results:**

The incidence of postoperative complications that demanded a hospital care was 24,09%. The main comorbidity presented in this sample was hepatic steatosis. The comorbidity that was associated with the postoperative period was type 2 diabetes. There was a tendency for the female gender be related to the complications. The cholecystectomy was the most frequent complication. Complications occurred during the first year in 57,35%.

**Conclusion:**

The most frequent complication was the need to perform a cholecystectomy, where the most frequent comorbidity was hepatic steatosis. Over half the complications occurred during the first year postoperatively. Type 2 diabetes was associated with the occurrence of postoperative complications; women had the highest incidence; body mass index was not associated with the occurrence of complications.

## Abstract (pt)

**Racional:**

A técnica cirúrgica atualmente considerada padrão-ouro para o tratamento cirúrgico da obesidade é o bypass gástrico em Y-de-Roux. No entanto, complicações podem surgir e o cirurgião deve estar preparado para elas.

**Objetivo:**

Avaliar retrospectivamente a ocorrência de complicações e fatores associados em pacientes submetidos à cirurgia bariátrica.

**Métodos:**

Analisaram-se 469 prontuários de pacientes e dados referentes às consultas ambulatoriais. As variáveis pesquisadas foram sexo, idade, estatura, índice de massa corpóreo pré-operatório, peso pré-operatório, comorbidades pré-operatórias, tempo de internação, complicações pós-operatórias que exigiram reinternação e o tempo decorrido entre o procedimento e a complicação. O seguimento dos pacientes foi de no mínimo um ano.

**Resultados:**

A incidência de complicações pós-operatórias que exigiram manejo hospitalar foi de 24,09%. A principal comorbidade foi a esteatose hepática. A comorbidade que se associou às complicações no período de pós-operatório foi o diabete melito tipo 2. Houve tendência do sexo feminino se relacionar às complicações. A colecistectomia foi a mais frequente complicação. Durante o primeiro ano ocorreram 57,35% das complicações.

**Conclusões:**

A complicação mais frequente foi a necessidade de realização de colecistectomia, sendo que a mais frequente comorbidade foi a esteatose hepática. Mais da metade das complicações ocorreram no primeiro ano de pós-operatório; o diabete melito tipo 2 associou-se às intercorrências pós-operatórias; o sexo feminino teve maior incidência; e o índice de massa corporal não correlacionou-se às complicações

## INTRODUCTION

The most effective available treatment for morbid obesity is the bypass surgery, which can result in quality of life improvement or complete morbidity resolution, even avoiding the beginning of new ones, making it possible to lose weight above 60% in a long run and decrease of mortality^2^^,^ 3. Besides, it shows low surgical rates and manageable index of complication, that fact can explain how its realization has spread^4^^,^ 18.

The actual gold standard technique for obesity treatment is the Roux-en-Y gastric bypass^8^. It shows good results, low mortality and low rate of adverse events^15^. However, it's important for surgeons to know the complications that may occur during the procedure and which features differ from the obese patients from the non-surgical ones, because it contributes for a better postoperative care^7^. The following are obese patients' features: 1) complications with minimal signs and physical symptoms; 2) hard to perform physical examination; 3) difficulty during clinical evaluation due to their body mass in gurneys and diagnostic exams; 4) the obese patient has little reserve to resist when them come down with serious diseases^11^. Their biotype and clinical limitations also provide technical obstacles to the radiologic studies that are regularly requested in the postoperative care period to look for complications^12^.

This way, it's possible to notice that the complications from the bypass surgery are increasing due to its biggest application as therapeutic method, and also due to hospital care difficult clinical characteristics that the patients who underwent bypass surgery may present. 

The objective of this study was to evaluate the complication occurrence in morbid obese patients who underwent surgical treatment.

## METHODS

This study was approved by the Research Ethics Committee of Santa Catarina University, under the following record number 19194113.5.0000.5369.

It is a cross-sectional epidemiological study, using secondary data base about patients who underwent bypass surgery in the city of Tubarão, SC, Brazil, between August 2006 and March 2013. The period in which the data was collected was between August 2013 and March 2014.

All the patients who underwent bypass surgery during the period described were included, using the hospital charts. Those patients who needed surgical re-intervention or hospital care were classified as complicated, even during the admission period for bypass surgery and as well as in postoperative care, the same complication might have happened without the need for hospitalization which was based on severity criteria. Besides hospital charts, the outpatient consultation records were also used. 

The exclusion criteria were patients who remained at the hospital or those who were readmitted for different reasons other than the bypass surgery, and also those who presented complication that didn't demand hospital stay.

The information collected was transmitted to a data record protocol, created by the authors, with the following variables: gender, age, height, preoperative BMI, preoperative weight, preoperative comorbidities, time of hospital stay, postoperative complications that demanded re-admission to the hospital and the time elapsed between the procedure and the complication. The complications were classified as immediate, premature and late. The immediate complications were those that occurred within four days after the procedure, the premature until 30 days and the late after 30 days.

The data was typed into an Excel 2010 program and the statistical analysis was done using the SPSS 20.0. The occurrence of the qualitative variables was described using relative and absolute frequency, whereas the quantitative variables were described with the mean and standard deviation. The Pearson's chi-squared test was used to verify the difference between the patients who had postoperative complications and those who didn't, confidence level of 95% (p<0,05).

## RESULTS

During the period of six years and seven months 469 bypass surgeries were evaluated. The ages varied between 18 and 67 years old (mean of 37,3±,26 years old). The female sex was predominant representing 363 patients (77,4%). The mean body mass index (BMI) was 41,6 kg/m^2^, standing between 28 and 63±5,31 kg/m². The admission period to perform the bypass surgery was an average of 3,84 days (2-23). 

**FIGURE 1:**
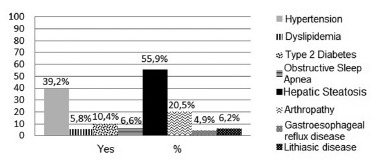
- Percentage of present comorbidities

The most frequent comorbidity found was hepatic steatosis (Figure 1) where the rest of the findings may be seen. The ultrasound report previous to the procedure wasn't available in 152 patients' charts.

**TABLE 1:** - Complication distribution in time according to its occurrence and percentage of total represented by each one of them

	**COMPLICATIONS**				
	**Immediate n(%)**	**Premature n(%)**	**Late n(%)**	**Absolute n (%)**	**Total %**
Fistulae	2 (22.22)	4 (18.18)		6 (4.41)	1.27
Bleeding	3 (33.33)	1 (4.54)		4 (2.94)	0.85
Stenosis	1 (11.11)	2 (9.09)	3 (2.85)	6 (4.41)	1.27
Abdominal pain	2 (22.22)	5 (22.72)	7 (6.60)	14 (10.02)	2.98
Superficial abscess	1 (11.11)			1 (0.73)	0.21
Profound abscess		3 (13.63)		3 (2.20)	0.63
Ulcer anastomosis		2 (9.09)	2 (1.90)	4 (2.94)	0.85
Necrotizing fasciitis		2 (9.09)		2 (1.47)	0.42
Pancreatitis		1 (4.54)	2 (1.90)	3 (2.20)	0.63
Deep venous thrombosis		1 (4.54)		1 (0.73)	0.21
Pneumonia		1 (4.54)		1 (0.73)	0.21
Internal hernia			5 (4.76)	5 (3.67)	1.06
Phytobezoar			1 (0.95)	1 (0.73)	0.21
Cholecystectomy			72 (68.57)	72 (52.94)	15.35
Nutritional deficit			5 (4.76)	5 (3.67)	1.06
Adhesions			2 (1.90)	2 (1.47)	0.42
Incisional hernia		22 (100)	6 (5.71)	6 (4.41)	1.27
TOTAL	9 (100)		105 (100)	136 (100)	28.99

Of those patients who underwent bypass surgery, 133 presented with complications and needed to be readmitted during the postoperative period, representing 24.09%. Considering the fact that some patients developed more than one complication, the amount of complications was 136 in that period, representing 28.99% of those surgical patients.

Although it is a complication that occurred only during late postoperative, the need to perform cholecystectomy was the most frequent complication, done in 15,35% of the patients. Listed on Table 1 are the complications and their representativeness.

**FIGURE 2:**
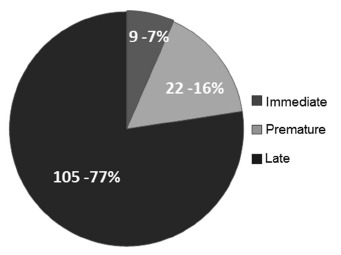
- Total percentage distribution of occurred complications according to the postoperative period

Figure 2 shows the percentage distribution of complications. Nine occurred during immediate period (6.56%), 22 during premature period (16.05%) and 105 during de late period (77.2). The mean time for the occurrence of complication was 339.8 days (+/- 335 days).

All of the patients were evaluated for at least one year postoperatively. Of those who had complications, 78 had them during the first year, representing 57.35%.

When studying the association between gender and complication occurrence, there was a significant tendency for the female (p=0.052). Regarding the association between any kind of preoperative comorbidities and the evolution to complication, there was no significant difference (p=0.388). Regarding the association between specific comorbidities and the evolution to complication, type 2 diabetes was the condition that presented significant difference (p=0.025). In Table 2 the remaining associations are showed.

**TABLE 2:** - Association between variables and complications

	**COMPLICATIONS**		
**VARIABLES**	**Yes n (%)**	**No n (%)**	**p**
Gender			
Female	95 (26.2)	268 (73.8)	0.052
Male	18 (17)	88 (83)	
BMI	112 (41.6)	349 (41.6)	0.937
Age	113 (38.3)	356 (36.9)	0.257
Hypertension	48 (26.1)	65 (22.8)	0.241
Dyslipidemia	5 (18.5)	108 (24.4)	0.331
Type 2 diabetes	18 (36.7)	95 (22.6)	0.025
Obstructive sleep apnea	8 (25.8)	105 (24)	0.481
Hepatic steatosis	71 (27.1)	42 (20.3)	0.054
Arthropathy	27 (28.1)	86 (23.1)	0.183
Gastroesophageal reflux disease	4 (17.4)	109 (24.4)	0.618
Lithiasic disease	8 (27.6)	105 (23.9)	0.650

All the surgical procedures done used the Roux-en-Y gastric bypass technique and there weren't any death events in the studied period.

## DISCUSSION

The evolution with complications during postoperative period of bypass surgery is more frequent related to type 2 diabetes. This event found in this study is consistent with the one done in London that mentioned that diabetic patients who underwent bypass surgery had an increased hospital admission risk^16^. Another showed that the diabetes was the most important mortality predictor factor^17^. Therefore, it is noticeable that the obese diabetic patient has a greater potential to have bad outcomes during the bypass surgery postoperatively. It might be explained by those patients' poor physiologic reserves that may be easily deteriorated when the body is exposed to physical stress.

In comparison, the evaluation between BMI and the occurrence of complication wasn't associated in this paper. A study conducted in Sweden found similar results^19^. Therefore, the results of this research and those other in literature point out that this is a weak risk indicator for complications of bypass surgery^14^.

Regarding the association between gender and the occurrence of complications, there was a tendency in the female to relate to a highest incidence. Wasn´t found in literature mention to this association, but due to the fact that the need for cholecystectomy was the main complication found in this study, the relation between the female with the complications can be explained, since women have independent risk for cholelithiasis. Besides, presuming that the need for cholecystectomy is relevant in this association, another possible explanation is that men have lower propensity to develop symptoms of lithiasic disease after bypass^10^.

In this study the complication rate was 28.99%, where 57.35% of them happened during the first postoperative year. Another study found inferior complication rate, 4.87%^20^. At the same time, the variables evaluated were distinct. A 15.6% complication rate was related, including immediate and late complications; this rate is close to the one found here^21^. So, there are differences in results describing the rates of complications due the differences in the variables considered, since there is no description about the most common complication in the postoperative period in those other studies. 

It is relevant to point out that obesity and fast weight loss are documented risk factors for cholelithiasis. Here, the lithiasic disease previous to the bypass surgery was evaluated (including asymptomatic cholelithiasis and cholecystectomy) as obesity comorbidity and, posteriorly, the need for cholecystectomy as a bypass surgery complication. Nevertheless, there is no way of knowing what motivated the cholecystectomy, if it was the previous cholelithiasis or the weight loss posterior to the surgery.

The second most often complication was idiopathic abdominal pain that was present in 2.98% of patients. This situation is important because it's the clinical presentation of a variety of diseases. The internal hernia, is the major reason to discard, due to its potential to complicate with incarcerated herniation, vascular suffering and need for resection. Studies describing this complaint as admission reason weren't found.

The nutritional deficiency requiring hospital admission occurred in 1.06%. According to the consulted literature, the presence of acute nutritional deficiency is rare, but it can be expected and needs premature diagnostics and effective treatment. The deficiencies are usually of fat-soluble vitamins and minerals, the most common are B12 vitamin, folate, zinc, thiamine (B1 vitamin), A vitamin and E vitamin. The acute Wernicke encephalopathy secondary to thiamine and B12 deficiency may induce permanent damage to the patient^9^.

As to the complications with endoscopic healing and diagnosis, the anastomotic ulcer was diagnosed in 0.85% of patients and it's inferior to the results of another research where they found 4%^21^. Stenosis is another complication in this group and happened in 1.27% of the sample, it was a postendoscopic treatment complication and the reason the patients were admitted.

Pancreatitis occurred in 0.63%, the same results weren't found in any other studies. All the cases that had this disease were from biliary cause, without evidence of choledocholithiasis imaging; in this case they were submitted to cholecystectomy.

Regarding the occurrence of complications of acute abdomen, internal hernias were found in 1.06% of patients who underwent bypass surgery and it's inferior to the numbers found in literature, 3.1% rate^1^. Adhesions were found in 0.42% of the patients in this research. In a study performed with a sample of 10 acute abdomen patients, three of them had adhesions as the cause, showing that it is an important cause of bowel obstruction^13^.

Bleeding occurred in 0.85% and it is also inferior to the numbers of other paper, 2%^21^. These might be intraperitoneal or intraluminal bleedings, the last ones are caused by the lines of suture, acute lesions or peptic ulcers. The clinical presentation is hypovolemic shock, caused by hemoperitoneum or digestive bleeding^6^.

From the total sample of this study, fistulae occurred in 1.27%, that was similar to the literature, 2.1%. This complication is important due to its high morbimortality, with sepsis inversely proportional to the prematurity of diagnosis and fistula treatment^7^.

In this study less frequent complications were found. Nevertheless, they must be known because it's important to identify them in the postoperative period for adequate care. They are: profound and superficial abscess in the operatory wound, necrotizing fasciitis, deep venous thrombosis, pneumonia, incisional hernia and phytobezoar. Abdominal pain, superficial abscess at surgical site and nutritional deficit, are diseases that occur separately, and they don't represent a superdiagnosis.

Some limitations exist in this research. The main was the use of information collected in secondary data base, in hospital charts, and clinical consultations charts, those are liable documents that may be described incorrectly, and also the post operative follow up period wasn't the same for every patient.

## CONCLUSIONS

The most frequent complication was the need for cholecystectomy, and the most frequent comorbidity was hepatic steatosis. More than half of complications happened during the first year of postoperative period; type 2 diabetes was associated with postoperative complications; women had a greater incidence; and the body mass index wasn´t related to the complications.
